# Virulence attenuation during an influenza A/H5N1 pandemic

**DOI:** 10.1098/rstb.2012.0207

**Published:** 2013-03-19

**Authors:** Maciej F. Boni, Tran Dang Nguyen, Menno D. de Jong, H. Rogier van Doorn

**Affiliations:** 1 Oxford University Clinical Research Unit, Wellcome Trust Major Overseas Programme, Ho Chi Minh City, Vietnam; 2 Centre for Tropical Medicine, Nuffield Department of Clinical Medicine, University of Oxford, Oxford, UK; 3 Department of Medical Microbiology, Academic Medical Center, University of Amsterdam, Amsterdam, The Netherlands

**Keywords:** influenza, H5N1, virulence evolution, mathematical modelling

## Abstract

More than 15 years after the first human cases of influenza A/H5N1 in Hong Kong, the world remains at risk for an H5N1 pandemic. Preparedness activities have focused on antiviral stockpiling and distribution, development of a human H5N1 vaccine, operationalizing screening and social distancing policies, and other non-pharmaceutical interventions. The planning of these interventions has been done in an attempt to lessen the cumulative mortality resulting from a hypothetical H5N1 pandemic. In this theoretical study, we consider the natural limitations on an H5N1 pandemic's mortality imposed by the virus' epidemiological–evolutionary constraints. Evolutionary theory dictates that pathogens should evolve to be relatively benign, depending on the magnitude of the correlation between a pathogen's virulence and its transmissibility. Because the case fatality of H5N1 infections in humans is currently 60 per cent, it is doubtful that the current viruses are close to their evolutionary optimum for transmission among humans. To describe the dynamics of virulence evolution during an H5N1 pandemic, we build a mathematical model based on the patterns of clinical progression in past H5N1 cases. Using both a deterministic model and a stochastic individual-based simulation, we describe (i) the drivers of evolutionary dynamics during an H5N1 pandemic, (ii) the range of case fatalities for which H5N1 viruses can successfully cause outbreaks in humans, and (iii) the effects of different kinds of social distancing on virulence evolution. We discuss two main epidemiological–evolutionary features of this system (i) the delaying or slowing of an epidemic which results in a majority of hosts experiencing an attenuated virulence phenotype and (ii) the strong evolutionary pressure for lower virulence experienced by the virus during a period of intense social distancing.

## 1. Introduction

Transmission of influenza A subtype H5N1 from poultry to humans has been a major public health concern since the emergence in 2002 and 2003 of H5N1 genotypes that are highly pathogenic in both humans and terrestrial poultry [1,2]. The most immediate risk arising from these avian influenza viruses is that they will evolve the capacity for sustainable human-to-human transmission, the potential evolutionary pathways of which have recently been described in a ferret model [3,4]. Sustainable human-to-human transmission significantly increases the probability of an influenza pandemic, and this risk has prompted the development of national and international pandemic preparedness plans over the past 10 years. Response strategies generally include minimizing general-population transmission through isolation/quarantine and social distancing (SD) measures, minimizing hospital transmission, use of antiviral drugs, and vaccination [5–13]. Pandemic preparedness plans can be evaluated only hypothetically, with mathematical models, but data are available from drug [14–16] and vaccine trials [17,18] as well as past pandemics [19–21] that help us narrow down the parameter ranges corresponding to the effectiveness of some of these interventions.

One aspect of pandemic preparedness planning that has been ignored is the potential of changing or evolving virulence during the course of the pandemic [22]. Although virulence can be measured in many ways, in this paper we focus on the case-fatality rate in humans as the main virulence phenotype of interest. Abundant evidence exists showing the potential for virulence evolution in both avian [23–27] and human [28,29] influenza viruses. However, it is not known how experimental evidence from animal models translates to infections in humans [30]. Empirical evidence for virulence evolution in influenza virus exists from the 1918 pandemic, whose case fatality attenuated from more than 2.5 to less than 0.1 per cent [31]. The potential for virulence evolution of H5N1 in humans is unknown.

A key feature of H5N1 infections that differs from previous influenza pandemics is the extreme case fatality in humans, currently estimated at 60 per cent [32]. It has been recently debated whether there are missing cases in the denominator of this calculation and whether the true case fatality of H5N1 infections is much lower than that observed from severe infections alone [33–38]; nevertheless, the current prevailing opinion leans towards this high 60 per cent estimate. This high case fatality makes it likely that virulence will attenuate during the course of a pandemic, but the more immediate question is whether a virus with this case fatality could successfully invade a human population and cause a sustainable outbreak. Therefore, in analysing virulence evolution of H5N1 in humans, we aim to describe two biological features of this system (i) the range of case fatalities that can sustain transmission in a human population and thus enable the evolutionary emergence [39,40] of H5N1 viruses and (ii) the pattern of virulence evolution during the course of the pandemic.

Classical evolutionary theory suggests that pathogens should evolve to be relatively benign, as killing the host has a negative impact on the pathogen's fecundity (transmission). In the 1980s and 1990s, a body of literature emerged explaining how a pathogen's evolutionary path to intermediate virulence—as opposed to zero virulence—could be caused by a positive association between transmissibility and virulence [41–45]. This work was later placed in the context of life-history trait evolution, and both the timing of and the correlation between transmission and virulence were identified as key elements determining a pathogen's evolutionary optimal virulence [46]. Non-equilibrium approaches were subsequently introduced to allow for the analysis of transient dynamics of virulence evolution [47,48], and these methods illustrate that virulence and transmission should evolve upwards during the upswing of an epidemic—when evolution places a premium on reproduction (transmissibility) over survival (infection duration)—and downwards during the downswing of an epidemic when survival is selected for more strongly than reproduction.

In this article, we construct a mathematical model of virulence evolution during an H5N1 pandemic using the quantitative-genetic strain structure introduced by Day & Gandon [48] and Boni *et al.* [49]. The clinical phenotypes of the different H5N1 strains in our model are based on data from known cases of human H5N1 infections [50–58], which often progress to severe infection, hospitalization and death. We build into the model two key phenotypic features of H5N1 viruses: their high replicative ability [59], and their high relative affinity for *α*_2,3_-linked sialic-acid receptors over *α*_2,6_-linked sialic-acid receptors [60,61]; both of these phenotypes are allowed to evolve in the model. In general, avian influenza viruses are adapted to *α*_2,3_ receptors, while human influenza viruses are adapted to *α*_2,6_ receptors. As the human upper respiratory tract (URT) contains predominantly *α*_2,6_ receptors, and the human lower respiratory tract (LRT) contains both *α*_2,3_ and *α*_2,6_ receptors [60–63], viruses evolving from *α*_2,3_ affinity to *α*_2,6_ affinity will simultaneously shift the viral burden from LRT to URT and become more infectious. In our model, the total viral burden in the LRT is positively associated with disease severity and case fatality [50,64]; thus, the phenotype of interest that we follow is the degree of LRT infection. In addition, we assume that infections with a higher LRT burden progress more rapidly to severity and death; hence, our model contains the same classic trade-off as previous virulence evolution models where higher mortality is associated with a shorter period of infectiousness [45,46].

The invasion of a virus across a species boundary is a much more difficult phenomenon to model, as this stochastic set of events is almost impossible to parametrize. Using estimates from the literature and, in the absence of data, the most reasonable ranges based on known aspects of the system's biology, we build a stochastic individual-based version of our mathematical model and test the success of viral invasion across a range of parameter values. These results are presented to give a general idea of the allowable range of invading case fatalities and the factors that influence this process.

## 2. Material and methods

### (a) Deterministic compartmental model

**Figure 1.**
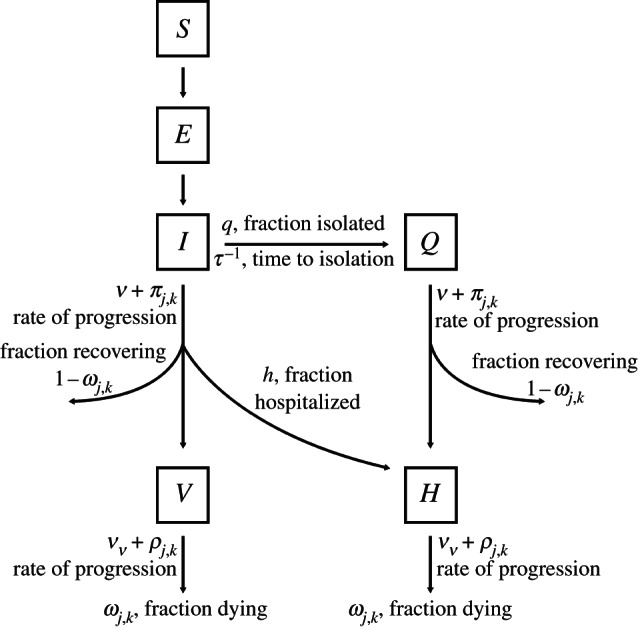
Class diagram for the model. Individuals can be susceptible (*S*), exposed (*E*), infectious (*I*), infectious with severe disease (*V*), isolated (*Q*) and hospitalized (*H*). Each infected individual is infected with a particular strain (*j*,*k*), which corresponds to a rate of progression and probability of recovery for that individual. Note the distinction between fractions and rates among the parameters.

We begin with a compartmental, deterministic differential-equations model, based on a classic *SEIR*-model in a closed population with no influx of additional susceptible hosts from other populations. Our model has additional classes for symptomatic individuals that have been placed under isolation (*Q*), severely infected individuals (*V*) and hospitalized individuals (*H*). Hosts in class *I* are infected and infectious. The basic flow diagram is shown in figure 1.

An evolutionary model is integrated into the basic population-dynamic model, as in previous models of evolutionary epidemiology [49,65], but with a two-dimensional phenotype space. Each strain in the model is indexed by *j* and *k*, with *j* = 1, … ,*J* and *k* = 1, … ,*K*. The index *j* describes the degree of affinity for *α*_2,6_ receptors, with increased affinity corresponding to an increase in *j*; as *j* increases the viral phenotype is better able to colonize the epithelium of the URT of humans and is more easily transmissible. The index *k* corresponds to the virus' intrinsic replicative ability, with higher *k* corresponding to higher replication. Influenza strains in phenotype space mutate via nearest-neighbour, reversible mutation; i.e. strain (*j*, *k*) can mutate to strain (*j*+1, *k*) or (*j*, *k*+1), but not both. Because only hosts and not individual viruses are followed in the model, a strain mutation corresponds to an individual host's entire viral population shifting, for example, from the (*j*, *k*) phenotype to the (*j*−1, *k*) phenotype. Thus, the mutation process describes the appearance of a new mutant and its rise to a high enough frequency that it will be the likely strain to be transmitted upon an infection event. These are the same assumptions as in other models of evolutionary epidemiology [48,49,65]. Hosts can only be infected with one viral phenotype at a time.

Once individuals become infectious, they enter the *I* class. A fraction *q* of *I* individuals will be placed under isolation (*Q*). Thereafter, a fraction *ω*_*j*,*k*_ of individuals in *I* and *Q* can progress to severe disease, and these individuals are classified as severely infected (*V*) or severely infected and hospitalized (*H*), respectively. All isolated patients progressing to severe infection are hospitalized, and a fraction *h* of *I* individuals progressing to severe disease are hospitalized. A fraction *ω*_*j*,*k*_ of severely infected individuals will die. Therefore, the strain-specific case fatality in the model is *ω*_*j*,*k*_^2^.

Note that the *ω*_*j*,*k*_ denotes both the fraction of infecteds that progress to severity and the fraction of severes that do not survive. As the future evolution of the severity phenotype of any pandemic influenza virus is completely unknown, we do not know if virulence attenuation will occur by lowering the probability that cases become severe, lowering the probability that severe cases progress to death, or both. For simplicity, we have assumed that these two probabilities are the same.

The basic transmission processes are governed by the parameters *α*_*j*_ and *β*_*k*_. The *α* parameters range from 0 to 1, and describe the degree of affinity for *α*_2,6_ receptors, and hence ability to colonize the URT and be transmitted; this is similar to the within-host parametrization used by Reperant *et al.* [66]. The parameter *α*_1_ is equal to zero, indicating that none of these strains is able to replicate in the URT, while *α*_*J*_ = 1, indicating that viruses of this phenotype are fully competent at invading the upper epithelium through *α*_2,6_-type receptors. Transmissibility is, therefore, directly proportional to *α*_*j*_. The parameter *β*_*k*_ is the basic transmission parameter in the model, which saturates non-linearly with *k* as2.1
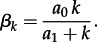


The force of infection of each strain is proportional to the product *α*_*j*_*β*_*k*_.

The parameters *ν* and *ν*_*v*_ represent the minimum recovery rates for infected individuals and severely infected individuals, respectively. Hosts in the *I* and *Q* classes progress to severity at rate *ν*+*π*_*j,k*_, where *π*_*j,k*_ is a parameter describing the increase in recovery rate for individuals infected with different strains in the model; *π*_*j,k*_ increases with *k* and decreases with *j* as infections with high replication in the LRT are the ones most likely to progress to severity quickly. Hosts in the *V* and *H* classes progress to death or recovery at rate *ν*_*v*_+*ρ*_*j,k*_; again, *ρ*_*j,k*_ introduces variation in the duration of severity for individuals with severe disease. The parameter *ρ*_*j,k*_ increases with *k* and decreases with *j*, as infections with high LRT burden are most closely associated with rapid progression to death. To simplify and focus the analysis, we define a variable describing the viral burden in the LRT as a fraction of maximal possible viral burden in the LRT. The variable,2.2
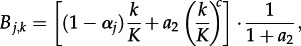
is bounded between zero and one; the expression *a*_2_ /(1+*a*_2_ ) is the fraction of receptors in the LRT that are of the *α*_2,6_ type, which in humans is the majority. The term (*k*/*K*)^*c*^ describes the increased LRT burden owing to high-replication phenotypes entering cells through both *α*_2,3_ and *α*_2,6_ receptors in the LRT. Note that when *α*_*j*_ = 1 and *k* is low, *B_j,k_* can be close to zero. In this situation, *B_j,k_* can be interpreted as the LRT burden caused by a low-replication human-adapted phenotype. The parameter *c* is unknown.

Finally, we introduce several necessary control parameters. The mutation rate is *μ*. Within-host viral populations can mutate one unit in phenotype space in either the *j* or *k* dimension; the mutation rate between any neighbouring pair of points in phenotype space is *μ*/4. A fraction *h* of infected hosts (not under isolation) will be hospitalized. A fraction *q* of symptomatic hosts will be isolated, with *τ*^−1^ being the time from infectiousness to isolation. *τ*^−1^ depends on both the public health measures taken to reduce transmission as well as the duration of pre-symptomatic transmission that would be occurring for a hypothetical H5N1 variant circulating among humans.

To write the full dynamical equations of the model compactly, we introduce two compound parameters, *γ_j,k_* and *f_j,k_*. The *γ* parameters describe how quickly hosts flow out of the *I* class; this rate is the harmonic mean of the recovery/progression rate and the isolation rate. We have
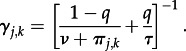


The dimensionless compound parameter *f_j,k_* describes the fractional flow of hosts from the *I* class to the *Q* class (note that *q* is the fraction of hosts that are isolated, but the fraction of the total flow out of *I* is higher, because isolation occurs more quickly than recovery or progression to severity). We have
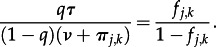


Note that 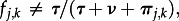 unless *q* = 0.5. The extra algebra is necessary as hosts can leave the *I* class at different rates. The full dynamical equations are2.3
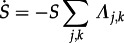
2.4
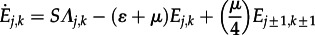
2.5
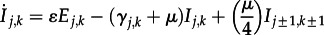
2.6
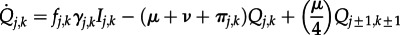
2.7
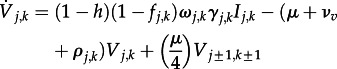
2.8
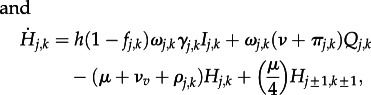
with the force of infection of virus (*j, k*) defined as2.7
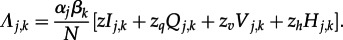
The *z* parameters describe the relative levels of mixing or circulation in the general population for the infected classes *I*, *Q*, *V* and *H*. Typically, we set *z* = 1. When SD is implemented, the parameters *z* and *z_v_* are reduced by a constant factor. *N* is the host population size.

The rates of disease progression, 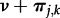 and 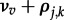, are two of the epidemiologically relevant phenotypes that will be evolving in the model. Both of these are linked significantly to the disease burden in the LRT. Hence, we define the relationships below
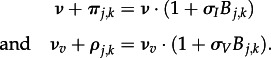
The parameters *σ*_*I*_ and *σ_V_* determine the amount of allowable variation for the recovery/progression rates in the model. The *ω* parameters are fractions that describe the proportion of individuals progressing from infection to severity, and from severity to death. In the flow from the *I* class to the *V* class, only a fraction *ω* progress to severity, while a fraction 1−*ω* recover. Note that if the flow out of the *I* class is 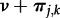 (assuming *q* = 0 for simplicity), the fraction recovering is not equal to 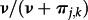.

**Figure 2.**
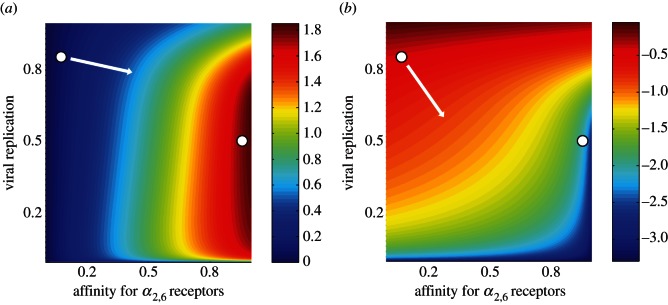
Contour plots in phenotype space showing (*a*) *R*_0_ value and (*b*) log_10_ case fatality for the different phenotypes in the model. The axes show affinity for *α*_2,6_ receptors (*j* index, horizontal axis) and level of viral replication (*k* index, vertical axis) and are scaled from 0 to 1. The basic reproductive number *R*_0_ is calculated from equations (2.3)–(2.8) (see the electronic supplementary material), and the case fatality is calculated from equation (2.9). The white circles in the top-left of each graph (*a,b*) show the approximate phenotypic position of current H5N1 viruses, low affinity for *α*_2,6_ receptors and a high level of viral replication. The white circles in the right of each graph (*a*,*b*) show the approximate phenotype position of seasonal human influenza viruses (i.e. subtypes H3N2 and H1N1); these viruses have a high affinity for *α*_2,6_ receptors and what we surmise to be an average level of viral replication. The white arrow in (*a*) shows the probable evolutionary path under the assumption that *R*_0_ is a good proxy for viral fitness. The white arrow in (*b*) shows the optimal evolutionary path from a public health perspective, i.e. the path that results in the most rapid virulence attenuation. *R*_0,max_ = 1.82.

The case fatality for a given virus is 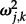, and the *ω* parameters are defined by2.9
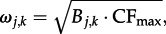
where CF_max_ is the maximum allowable case fatality in the model. Thus, we assume that case fatality is directly proportional to LRT burden. Figure 2 shows the case fatality and *R*_0_ values in phenotype space, the optimal evolutionary path of the virus, and the optimal evolutionary path from a public health perspective. In these two phenotype-space maps, we see that *α*_*j*_ has the biggest impact on the invading virus's *R*_0_, and thus that evolution should favour more rapid change in *α*_*j*_ than in *β*_*k*_. From a public health perspective, evolutionary change in both the *α*- and *β*-dimensions would be optimal as this would result in the most rapid reduction in case fatality. The white arrows in these graphs show, in phenotype space, the directions of maximal change in *R*_0_ and maximal change in case fatality.

### (b) Stochastic individual-based model

Details of the implementation of a stochastic individual-based model, paralleling equations (2.3)–(2.8), are contained in the electronic supplementary material.

### (c) Parameters

Unless otherwise noted, the following parameters were used for simulations. Phenotype space has *J* = 10 evenly spaced classes for receptor specificity [67], and *K* = 40 classes for viral replicative ability. The maximum *R*_0_ in phenotype space was set to *R*_0,max_ = 3.5; when this parameter is changed, all *R*_0_ values for the 400 phenotypes in phenotype space are rescaled by the same amount. For variation in infection/severity durations, we set 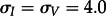. Time from infectiousness to isolation *τ*^−1^ was set to 2.2 days, with 2 per cent of all cases being isolated (*q*). The hospitalization fraction *h* was set to a constant value of 0.50, indicating that the pandemic did not overwhelm the health system. The parameter *a*_2_ was set to 1.75, corresponding to 64 per cent of epithelial cell receptors in the LRT having *α*_2,6_ linkage; this parameter is unknown [68]. Other parameters are set as in table 1.

**Table 1.** Model parameters. N/A designation is used for *α* and *β* parameters that span all of phenotype space as well as compound parameters.

parameter	description	assumed value/range	evidence
*α*_*j*_	affinity for *α*_2,6_ receptors of strain (*j,k*); 1−*α*_*j*_ is interpreted as the affinity for *α*_2,3_ receptors	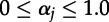	N/A
*β*_*k*_	transmissibility parameter for strain (*j,k*)	defined by 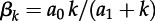	N/A
*a*_0_, *a*_1_	transmission saturation parameters for strain with replicative ability *k*	*a*_1_ = 4.0; *a*_0_ set such that 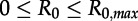	none
*R*_0,max_	maximum possible *R*_0_ value in phenotype space	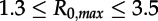	none
*μ*	mutation rate	0.05	none
*ν*, *ν*_v_	minimum recovery rates for infected and severely infected individuals, respectively	*ν*^−1^ = 9.0 days	[50–58]
		*ν*_*v*_^−1^ = 15.0 days	
*σ*_*I*_	amount of phenotypic variation in the progression rate of infected individuals (*I* and *Q*); progression rate can vary 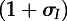 -fold from minimum rate *ν*	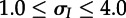	[50–58]
*σ*_*V*_	amount of phenotypic variation in the progression rate of severely infected individuals (*V* and *H*); progression rate can vary 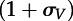 -fold from minimum rate *ν*_*v*_	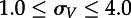	[50–58]
*a*_2_	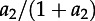 is the fraction of receptors in the LRT with *α*_2,6_ linkage	1.75	none
*c*	concavity parameter describing effect of high viral replication on LRT burden *B*_*j,k*_	10	none
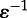	duration of exposed but uninfectious period	1.4 days	[69,70]
*τ*^−1^	days from infectiousness to isolation	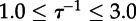	[71,72]
*q*	fraction of all cases that are isolated; will vary widely depending on virulence and context	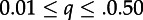	[72]
*h*	fraction of non-isolated cases that will be hospitalized upon progression to severity; will vary widely depending on public health system	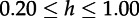	none
*z_q_*, *z_v_*, *z_h_*	relative mixing levels in population of isolated, severe and hospitalized individuals, respectively	*z_q_* = z*_h_* = 0.05 *z_v_* = 0.80	none
CF_max_	maximum case fatality across all strains	0.90	none
*γ*_*j,k*_	compound parameter describing the rate at which individuals flow out of the *I* class	defined by 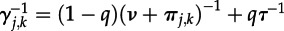	N/A
*f_j,k_*	compound parameter describing the fractional flow of hosts from the *I* class to the *Q* class	defined by 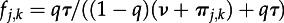	N/A

## 3. Results

### (a) Theory

To simplify analysis of the dynamical system in equations (2.3)–(2.8), we set *μ* = 0 and remove the exposed class (*E*), so that the dynamical equation for *I_j,k_* has the form 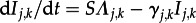. We use the uppercase variable *X* to describe infected individuals of any severity or hospitalization/isolation status: *X* = *I* + *Q* + *V* + H and 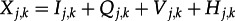. We will write down the dynamical equations for the strain frequencies
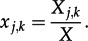
For convenience, we define 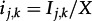, 
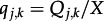
, 
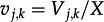
 and 
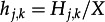
, so that
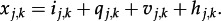
We will denote the sums of the frequencies in the four different infected classes by lowercase boldface variables, e.g. 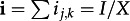. We have 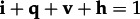.

We use the uppercase variable *Y* to denote the mixing-weighted number of infected individuals: 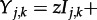 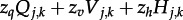, and we define 
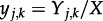
. Then, 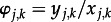 represents the proportion of any given strain that is currently in circulation; 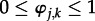.

Using these frequency variables above and making the appropriate substitutions from equations (2.3)–(2.8), we can write down
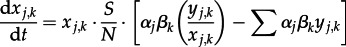

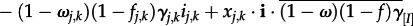

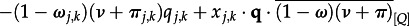
3.1
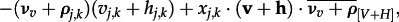
where the bars indicate means, and the bracketed subscripts, e.g. [*Q*], show that means are taken over only certain population classes.

From the first line, we see that the product *αβ* should always evolve upwards. When all the *z* parameters are equal to one, the expression in the square brackets of the first line of (3.1) reduces to 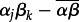, corresponding to the standard population-genetic result that a strain's frequency will increase if its *αβ*-value is larger than the mean *αβ*-value in the population at the time. From the second and third lines of the above equation, we see that there is selective pressure on the ‘progression to severity’ parameters, (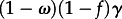 and 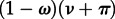), to evolve downwards. Because there is a negative sign in front of the first term on each line (which describes the strain-specific progression rate from infection to severe infection) and a positive sign in front of the second term (the mean progression rate across individuals in a particular population class), a strain whose progression rate is faster than average will be selected against as these two terms will add to a negative value in the 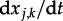 equation. Therefore, the two progression rates evolve downwards, and *ω* is under selection to evolve upwards. However, *ω*_*j*,*k*_ is also positively correlated with *π*_*j,k*_ and *γ*_*j,k*_, which places it under selection pressure to evolve downwards. Thus, a complex set of selection pressures operates in the *I* and *Q* classes of our structured model. In these two classes, selection favours more severe phenotypes because a higher probability of progressing to severity prolongs the infection; however, selection also acts against more severe phenotypes because they are more likely to induce patient death and shorten the infectious period. The fourth line can be interpreted the same way as the second and third lines. However, because there is no (1−*ω*) term on the fourth line, *ω* is under pressure to evolve downwards as this extends the duration of infection in these classes and reduces the probability of death. Note that when means and frequencing are computed only for certain population classes, this indicates that evolution is being driven by competition among viral phenotypes in only these classes of hosts.

To track the evolution of *ω*, the square root of the case fatality, we define3.1
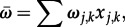
and we have3.2
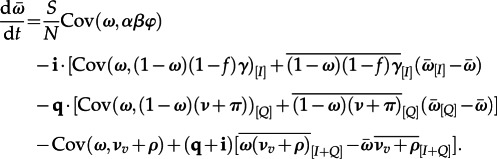


The dynamical equation above is written in the classical structure of the Price equation [73,74], with the first line showing that the covariance between a case-fatality proxy (*ω*), and a transmission proxy (*αβφ*) is the primary driver in the evolutionary dynamics of virulence in the system. The covariance between *ω* and *α* is negative, whereas the covariance between *ω* and *β* is positive, meaning that the term on the first line can push the case fatality in either direction; this will depend on which of these two characteristics currently has more phenotypic variation in the population. Note that *φ* will be a scalar value when the mixing parameters in the four different infected classes are equal.

On the fourth line of equation (3.2), the positive covariance between *ω* and *ν_v_* + *ρ* indicates that the association of rapid disease progression and high case-fatality should drive case fatality down in the long term (classical trade-off). However, note that the term on the second half of this line dampens this effect because rapid progression in the *I* and *Q* classes is associated with a lower probability of recovery, and can be associated with a prolonged total infection time. Thus, selection pressures in this model are highly structured and operate differently in different clinical states. When we consider hosts in only the *I* and *Q* classes (early phase of infection), prolonged infections can be associated with both higher and lower severity, complicating the effects of the classical trade-off between virulence and infection duration. Assuming mixing and shedding patterns do not change during the course of an infection, the evolutionary optimal behaviour of the virus is to prolong the infection period without increasing the case fatality, but the relationship between duration and severity is not monotonic. Thus, life-history characteristics of the virus are critical for analysis of a multi-stage infection with varying degrees of severity [46]. The non-monotonic relationship between duration and severity is seen clearly on the second and third lines of equation (3.2), where the (1−*ω*) terms show that higher case fatality can be associated with longer durations of infection.

The main evolutionary–epidemiological effect to note in equation (3.2) is the product of the number of remaining susceptibles *S*/*N* and the covariance between case fatality and transmissibility. In the early phases of the epidemic, this interaction will have a strong effect on positively selecting for higher transmission; as the epidemic progresses, the strength of this effect will wane and strains with longer infection durations will be selected. The second effect we expect to see is a bottleneck effect, if the parameter *φ* is suddenly reduced through a public health intervention such as SD; if the reduction in *φ* is large, selection pressures for longer viral survival and lower virulence will intensify.

### (b) Criteria for invasion

**Figure 3.**
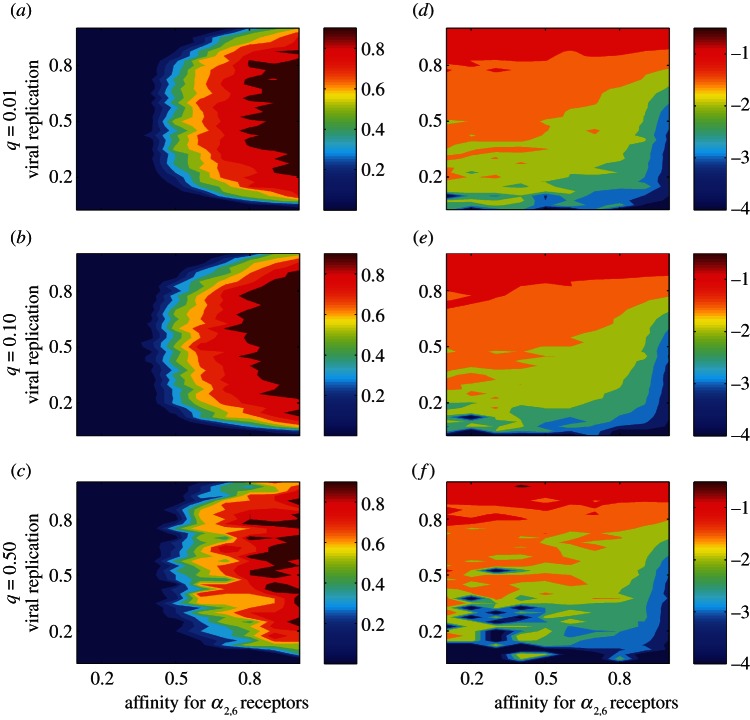
Contour plots in phenotype space showing (*a*–*c*) probability of successful invasion and (*d*–*f*) median log case fatality computed across successful invasions only. Axes as in figure 2. Each point (*j,k*) corresponds to 100 stochastic simulations. Each row of plots corresponds to a different level of isolation (*q*). (*a*–*c*) make clear that only certain phenotypes are able to invade, (*d*–*f*) show that these phenotypes correspond to a wide range of case fatalities. Mutation rate *μ* = 0.05.

The ability of H5N1 phenotypes to invade was assessed with an individual-based stochastic model in a population of one million individuals. Figure 3 shows how often invasions were successful for different phenotypes in (*j, k*) space. The main determinant for invasion is the parameter *α*_*j*_, describing a strain's ability to colonize the human URT. In addition, viruses with low replication (low *k*) cannot invade owing to poor transmissibility, and viruses with very high replication (high *k*) are poor invaders owing to their association with rapid progression to severity and death. Successful invaders are associated with a wide range of case fatalities, illustrating a core problem in attempting to determine the case-fatality phenotype of a successfully emergent H5N1 variant in humans.

In figure 3, 40 000 simulations were run for each plot, 100 for each (*j, k*) pair. Approximately 16 000 simulations resulted in successful invasions for realistic levels of isolation (*q* = 0.01 and *q* = 0.10), and 2635 simulations resulted in successful invasions when the isolation fraction was set to 50 per cent, an unattainable level in most public health contexts. The median invading case fatality for *q* = 0.01 and *q* = 0.10 was 5.5 per cent (IQR 2.5–11.1%), and the median invading case fatality for *q* = 0.50 was 5.7 per cent (IQR 2.6–12.1%). This suggests a small effect of isolation fraction on the early phases of virulence evolution. More detailed study is needed on this topic as it is also likely that ability to isolate patients will be correlated with clinical presentation and severity, a feature that is not included in our model.

To determine which other parameters correlate with probability of successful invasion a sensitivity analysis [75] was done on 13 parameters, including the initial values of *j* and *k* for the starting viral phenotype (for details see the electronic supplementary material). Partial rank correlation coefficients were computed between the parameters and the probability of a successful invasion. The initial value of *j* (PRCC = 0.875) and the value of *R*_0,max_ (PRCC = 0.830) had the largest effects on this probability. As figure 3 suggests, a higher *j* value for an initial invading strain will have a large effect on generating a successful epidemic. Likewise, increasing the *R*_0,max_ has a large effect as this inflates the *R*_0_ values in all of phenotype space and makes it more likely that a mutation will find a phenotype with *R*_0_ > 1. The parameter with the third largest effect was the mixing rate *z_q_* = *z_h_* for isolated/hospitalized individuals (PRCC = 0.197), showing that the effects of severity, hospitalization and reduced mixing play an important role in determining if a particular phenotype will have the right characteristics to invade.

For the simulations that do result in a successful invasion, we computed the ‘invading case fatality’ by recording the mean case-fatality phenotype after the epidemic had reached 1000 cases. The invading case fatality correlated most strongly with the initial value of *k* (PRCC = 0.957) and the convexity parameter *c* (PRCC =− 0.824). Probability of invasion is highly dependent on a virus being able to enter epithelial cells through *α*_2,6_ receptors; therefore, for an invading virus to be associated with a high case fatality, it must also be a fast replicator to induce a high viral burden in the LRT through *α*_2,6_ receptors in the LRT. For this reason, we see strong partial correlations with the parameters *k* and *c*, as these parameters determine the ability of a URT-adapted virus to induce significant viral burden in the LRT. The partial correlation with *c* is negative because strong convexity means that only the most rapid replicators maximize the LRT burden. The next strongest partial correlation was with the parameter *a*_2_ (PRCC =−0.183) which describes the abundance of *α*_2,6_ receptors in the lower respiratory tract. Likewise, this parameter describes an *α*_2,6_-adapted virus's ability to induce significant burden in the LRT. Taken together, these PRCC values mean that when *α*_2,6_-adapted viruses are able to induce a significant LRT burden, the case fatality associated with an invading virus should be high. All PRCC values are plotted in the electronic supplementary material, figure S2.

### (c) Virulence dynamics and social distancing

**Figure 4.**
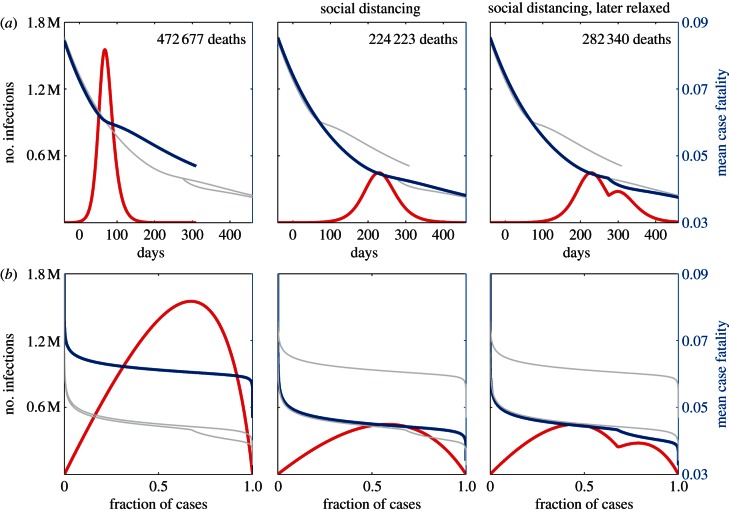
Example of effects of social distancing (SD), simulated from equations (2.3)–(2.8) in a population of *N* = 10^7^ individuals, showing infected individuals (red) and mean case fatality phenotype (blue). The left panels show pandemic progression with no SD. The middle and right panels show a SD policy, triggered at 100 cases, that reduces contact rates by one third. In the right-hand panels, the SD policy is relaxed on the downswing of the pandemic and contact rates return to their previous levels. (*a*) Show pandemic dynamics in time. (*b*) Show pandemic dynamics on the ‘suscetpibles’ axis, (*N*−*S*(*t*))/(*N*−*S*_final_), describing the fraction of individuals that have already been infected. The grey lines show the case-fatality curves from the other two panels. (*b*) Show that the majority of individuals experience the same case-fatality phenotype as the epidemic dynamics are fast relative to the evolutionary dynamics. The middle panels show that the main beneficial effect of SD comes from delaying peak epidemic dynamics (DEL) which, in these simulations, allow the case fatality to attenuate from 6.0 to 4.5%.

As expected from the model construction and parametrization, virulence attenuates in the model. Figure 4*a* (left) shows a typical epidemic curve with *R*_0,max_ = 2.5, with case fatality attenuating from approximately 7 to 6 per cent during the epidemic. The other panels in figure 4 show the effects of an SD policy that lasts the entire duration of the epidemic (middle panels) and an SD policy that is relaxed when incidence has begun to decrease (right panels). The first consequence of SD is that peak epidemic dynamics are delayed, giving the virus more time to attenuate and resulting in a lower *per capita* case fatality during the epidemic wave. In theory, every SD policy will introduce a delay (DEL) in epidemic dynamics, and an SD policy with the right level of contact reduction implemented for the right amount of time may drive the system close to the epidemiological optimum (EPO) of infecting exactly 1−1/*R*_0_ individuals without over shooting (the ‘soft landing’) [19,20]. If the epidemic has not removed enough susceptible hosts from the population, a second epidemic wave will occur if SD measures are relaxed too early; this effect is shown in the right-hand panels of figure 4. The intermediate SD policy that guides the system to the EPO is more optimal than a stronger SD policy that extinguishes the epidemic in its early phases, as the strong-SD strategy will leave too many susceptibles in the population (*R*_effective_ > 1), making the population vulnerable to a reintroduction of the virus and a delayed but full-sized epidemic wave.

Although the blue virulence curves in the top panels of figure 4 look somewhat similar in time, they are experienced completely differently by the host population because an SD policy delays the epidemiological dynamics in relation to the evolutionary dynamics. Figure 4*b* displays evolutionary dynamics on the ‘susceptibles’ axis instead of the time axis, showing that the virulence levels experienced by the majority of infected people are very different when SD is implemented. Note that the virulence curves show ‘kinks’ or ‘cusps’ when there are abrupt changes in transmission or mixing, either due to implementation/relaxing of an SD policy or the natural dynamics of the system. This is a result of the complex effects of transmission, mixing and infectivity parameters on pathogen evolution which act both to (i) change selective pressures and (ii) alter the rate of evolution [49].

In addition to the observed DEL and EPO behaviours, the system may have an evolutionary optimum (EVO), whereby a SD policy with an extremely high contact reduction forces the viral population through a bottleneck and drives the virulence down very quickly. A sudden extreme reduction in contact rates acts to immediately reduce the effective size of the susceptible population, possibly lowering the reproduction number *R* below 1; as a result, each virus has difficulty finding a new susceptible host and selection strongly favours longer viral survival which is correlated with lower case fatality.

**Figure 5.**
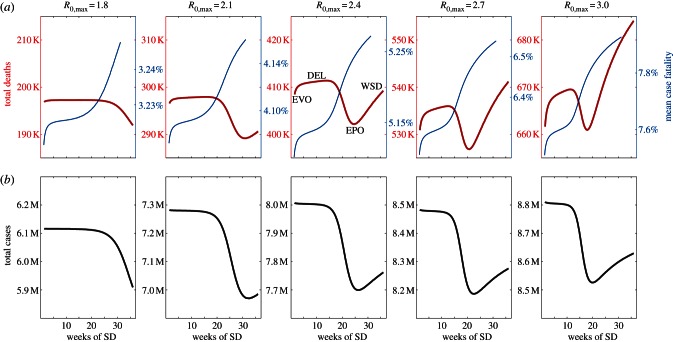
Effects of SD policy, using equations (2.3)–(2.8), when the product duration × intensity is fixed. The number of weeks of SD (*w*, shown on horizontal axis) and the percentage reduction (*p*) are constrained by *pw*= 10/7; hence, a 5-week SD policy corresponds to the mixing parameters (*z*, *z_v_*) being reduced by 2/7, 5/7 of their original value. (*a*) The red lines show the total number of deaths in a population of 10 million individuals, and the blue lines show the mean case fatality experienced by the population. (*b*) The black lines show the total number of infections during the epidemic, and the transition from DEL to EPO is easily observed in these graphs. In the middle panel, four different behaviours are labelled describing the non-monotonic relationship between SD duration and number of deaths in the epidemic. EVO and EPO mark the evolutionary and epidemiological optima, respectively. When SD is not intense enough to significantly drive down the virulence phenotype (EVO) and not sustained long enough to allow for a soft landing (EPO), SD acts to delay the epidemic (DEL), but not alter its size or duration; the delay results in the population experiencing a somewhat attenuated virulence phenotype. Note that *per capita* case fatality is higher under EPO than EVO, but the total number of cases is lower under EPO. When a low contact-rate reduction is implemented for a long time, we observe a weak effect of social distancing (WSD) and no significant effect on epidemic dynamics or virulence evolution.

The EVO can be seen in figure 5, where we consider different durations of SD, with longer SD durations having more modest reductions in contact rates. In each scenario, we consider *w* weeks of SD with host contact rates reduced by a percentage *p*, and we keep the product *pw* constant. The analysis reveals two local minima for the number of deaths during the course of the epidemic, and these are marked by EVO and EPO in the middle panel of figure 5. In between the EVO and EPO behaviours, an SD policy is not intense enough to drive down the virulence phenotype and it is not sustained for long enough to guide the epidemic to a soft landing; in this situation, we simply have a delayed full-sized epidemic (DEL). Varying the maximum *R*_0_ in the model shows that the relative number of deaths at the EVO and EPO optima can change quite dramatically. For low *R*_0_, a public health strategy aimed at an epidemiological soft landing (EPO) is the better choice as there is very little bottlenecking effect from intense SD. For high *R*_0_, the bottlenecking effect is quite strong, while aiming for a soft landing risks implementing an overly weak social distancing (WSD) policy resulting in almost no reduction in cases or deaths.

## 4. Discussion

Our study is intended to be read as a theoretical treatment of influenza's possible evolutionary pathways during an H5N1 pandemic. To the best of our knowledge H5N1 virulence evolution models in humans have not been analysed before, and our initial investigation of this topic led us to a problem that required the integration of epidemiological modelling, quantitative genetics and clinical science. We were able to take advantage of a growing literature of quantitative-genetic epidemiological models [48,49,65,76], but had to extend the analysis in these models from one-locus to two-locus systems. Despite the obvious benefits of being able to understand virulence evolution and its relationship to public health interventions, this and other models designed to analyse H5N1 virulence evolution have major limitations, because we have never seen an H5N1 variant successfully circulating among humans. We do not know if the means and ranges for infection duration will be the same as those we have observed in previous human H5N1 cases. We do not understand whether we have a linear or nonlinear effect of viral replication on LRT burden (parameter *c*). We do not know the number of different phenotypic variants we expect to see during a period of human adaptation and virulence evolution. We have no information on the mutation rates among these hypothetical phenotypes. And, we do not know the relative values of the mixing parameters for severely infected individuals or isolated/hospitalized individuals. All of these aspects of virus genetics, clinical presentation and human behaviour are critical to determining the evolutionary path of a human-adapted H5N1 virus. We have chosen the most plausible estimates and ranges in the analysis presented here, but much uncertainty remains.

One strength of our model is that the clinical design is based on severity patterns, infection durations and hospitalization times of past H5N1 infections. These clinical phenotypes were linked to what we believed were the two key causative viral phenotypes: the overall viral replication rate and the affinity for *α*_2,3_ and *α*_2,6_ receptors in the human URT and LRT. The observed clinical phenotypes of H5N1 infections over the past decade seemed to have normal progression to severity, rather than rapid progression to severity, indicating possibly that *σ*_*I*_ ≪*σ*_*V*_. In addition, one feature of H5N1 infections that was not included in our model was the observation of a significant number of recovering cases after a long period of severe disease. The current model formulation assumes the classical trade-off from the theory of virulence evolution, that virulent phenotypes are associated with more rapid progression to both death and recovery. This is important because in classical models, virulence attenuates because virulent phenotypes are associated with shorter durations of infectiousness. The known clinical pattern of H5N1 cases, however, is closer to variable progression to death and slow progression to recovery. When recovery is slow, low case-fatality phenotypes would have an even greater selective advantage, but only if we were to assume that viruses continue to be transmitted throughout the whole course of severe disease. However, it is not clear if these prolonged recovery periods will be observed in a human-adapted H5N1 virus, as a partial switch in receptor preference that establishes human-to-human transmissibility may also be associated with the normal recovery patterns seen in URT influenza infections.

The timing of transmission during the course of an infection is an epidemiological variable not included in the current model. For non-H5 human influenza cases, transmission is believed to occur mainly in the early stages of infection [77], suggesting that human-adapted influenza viruses should not experience a fitness cost from high virulence. For human-adapted H5N1, it is impossible to say at this stage what the pattern of shedding or transmission would be, but reproduction in the respiratory tract may occur longer than for typical human influenza viruses [57]. Isolation of virus from stool in past severe cases suggests an additional non-respiratory route for transmission [51,52].

In our analysis, successful viral invasion in both the deterministic and stochastic simulations did not occur for case fatalities close to 60 per cent. A large majority of invading viruses in the simulations were associated with case fatalities below 15 per cent, suggesting that the current circulating viruses may be too virulent to spread in human populations. One recent paper showing adaptation and mammal-to-mammal transmission in a ferret model is consistent with this finding, as it demonstrated lower case fatality after initial adaptation [3]. In light of this, the current situation in Egypt where a unique clade causes sporadic infections in humans with a significantly lower case fatality (36%, *n* = 168) than observed in Indonesia (83%, *n* = 190) and Vietnam (50%, *n* = 123) is worrying [32], although this difference in case fatality may be caused by earlier implementation of treatment.

The public health benefit of understanding virulence evolution is clear. Knowledge of the interaction between epidemiological and evolutionary dynamics helps us assess the potential outcomes of public health interventions. The soft landing EPO described here and elsewhere [19,20] is the ideal intervention in the absence of any evolutionary effects. The possibility of virulence evolution creates a second optimum (EVO) at high *R*_0_ values, where intense SD selects very strongly for longer-surviving low-virulence viruses and allows the majority of the population to experience the low-virulence phenotype. If both factors are being taken into account, a decision must be made whether resources will be focused on maintaining modest SD for a longer period or implementing more intense SD for a shorter duration—an influenza holiday—during which time the virus could attenuate significantly or go extinct. In a connected world, this later strategy may be quite risky. For a pathogen as serious and deadly as H5N1, perhaps more public health resources should be allocated to make SD policies both long and intense. Self-induced SD, potentially induced by panic, should also be considered when evaluating public health response in pandemics [78].

Despite the potential public health benefits, there is currently not enough data to warrant giving detailed advice in a scenario of an emerging or circulating H5N1 virus. Better evidence can be gathered through further animal experiments that have so far showed that (i) H5N1 needs few mutations and few cycles of infection to become transmissible in mammals [3,4] and (ii) adaptation to mammals was associated with a favourable clinical outcome for the host [3]. These and additional data coming from further animal and *ex vivo* cell culture passaging experiments should give incentive for continued sampling of the animal reservoir, and of human cases to monitor the emergence of these and other mutations. In addition, these results and our model suggest that careful monitoring of the clinical phenotype in sporadic and clustered cases combined with sequencing of viruses is especially important, but unfortunately more difficult, in clinically less severe cases as that is the expected phenotype of an adapting virus. Continuation of sequencing of human and animal isolates, repeat seroepidemiological studies in high-risk cohorts in endemic areas, and active human case-finding around animal outbreaks will be required to provide more information on the true range of phenotypic variation of human H5N1 cases.

Finally, models will need to be extended to include the geographical structure that is critical to analysing accurate and representative pandemic scenarios, as this allows us to obtain a realistic set of timings and delays for the pandemic in different locations. Modelling pandemic spread among different countries will also tell us the benefit associated with a certain public health strategy when other countries or regions are pursuing a different strategy [79]. For example, a precisely-timed SD policy may have little effect if neighbouring countries have not implemented anti-pandemic measures and are continually exporting viruses. Relaxing SD policies can be dangerous in this context. In addition, migration is likely to be associated with low virulence, resulting in the selection of lower virulence phenotypes seeding epidemics in new locations. As this serial bottlenecking could have a beneficial public health outcome, modelling efforts should focus on analysing response strategies that can take advantage of or perhaps amplify this effect. In addition to global geographical structure, local/city structure should be added to pandemic virulence models, as intervention strategies will undoubtedly focus on schools and hospitals.

Models on potential H5N1 virulence evolution will also need to look at the effects of drugs and vaccines [10–12], especially because antivirals can shorten the transmission period and select for a certain range of viral phenotypes [80]. Natural immunity should also be considered as the background *in vivo* immunity against other influenza viruses by humoral and cellular immune responses will have an effect on shedding patterns [66], invasion, pandemic progress and evolution. Host and age factors should also be considered for these types of model as some types of individual may experience influenza more severely than others. The most critical missing piece for which we would need to obtain data is the structure of phenotype space and the range of clinical presentations and transmissibilities therein. With no data describing the basic viral fitness landscape, we are still at a stage of making conjectures about the adaptation of H5N1 viruses to sustained transmission among humans.
